# Dietary rosemary oil with/without zymogen forte improves water quality, growth hormones, immune-physiological response, stress resilience, and health status of *Chelon ramada* grown in groundwater

**DOI:** 10.1186/s12917-024-04446-5

**Published:** 2025-01-17

**Authors:** Ashraf I. G. Elhetawy, Mohammed F. El Basuini, Ahmed I. A. Mansour, Shimaa A. Shahin, Amira A. Omar, Mohamed M. Zayed, Mohamed M. Abdel-Rahim

**Affiliations:** 1 Aquaculture Division, National Institute of Oceanography and Fisheries, NIOF, Cairo, Egypt; 2 Faculty of Desert Agriculture, King Salman International University, South Sinai, Egypt; 3 Animal Production Department, Faculty of Agriculture, Tanta University, Tanta, Egypt; 4 Animal and Fish Production Department, Faculty of Agriculture, Saba-basha, Alexandria University, Alexandria, Egypt; 5 Department of Fish diseases and Management, Faculty of Veterinary Medicine, Kafrelsheikh University, Kafrelsheikh, Egypt; 6 Department of Aquaculture, Faculty of Aquatic and Fisheries Science, Kafrelsheikh University, Kafrelsheikh, Egypt

**Keywords:** *Chelon ramada*, Rosemary, Zymogen, Brackish groundwater, Pathogens control, Immunogenicity, Heat shock proteins 70 and 90, Stress markers, Welfare

## Abstract

With freshwater resources becoming scarce worldwide, mariculture is a promising avenue to sustain aquaculture development, especially by incorporating brackish and saline groundwater (GW) use into fish farming. A 75-day rearing trial was conducted to evaluate fish growth, immune response, overall health, and water quality of *Chelon ramada* cultured in brackish GW and fed on a basal diet (BD) augmented with rosemary oil (RO) or RO + zymogen forte™ (ZF) as an anti-flatulent. Five treatments were administrated in triplicate: T1: fish-fed BD without additives (control group); T2: fish-fed BD + 0.5 g RO /kg diet; T3: fish-fed BD + 0.5 g RO and 1 g ZF /kg diet; T4: fish-fed BD + 1 g RO /kg diet; T5: fish-fed BD + 1 g RO and 1 g ZF /kg diet. Three hundred fish (8.51 ± 0.01 g/fish) were housed in 15 fiberglass tanks (1500-L tank). The results revealed significant improvements (*P* < 0.05) in growth performance, survival, growth hormone, and insulin-like growth factor-1. Additionally, there were decreases in the feed conversion ratio (FCR) and the levels of nitrogen by-products (NH_4_, NH_3_, and NO_2_) and pathogenic bacterial counts in the rearing water when fish were fed diets supplemented with RO and RO + ZF. Furthermore, significant reductions in the levels of plasma stress indicators (cortisol, creatinine, and glucose) were detected. In addition, there were significant enhancements observed in the levels of innate immune markers, such as white blood cells, total protein, albumin, and immunoglobulin. The complement system, specifically complement 3 and complement 4, also showed considerable improvements. Furthermore, there were increases in plasma heat shock proteins HSP70 and HSP90, as well as enhanced antioxidant activity. These gains were associated with healthier liver and intestines. The investigation demonstrated that adding 0.5–1 g RO / kg diet or RO + ZF to a *C. ramada* diet has many benefits, including reducing the levels of nitrogen by-product chemicals and pathogenic bacterial load in GW used in growth tanks. Furthermore, significant improvements were observed in the rates of growth and associated hormones, efficiency of feed utilization, blood indicators, immune function, condition of internal organs (namely the intestine and liver), and overall health of the fish.

## Introduction

The aquaculture of mullets (*family Mugilidae*) has increased in many regions across the globe, particularly in the Middle East and South Asia. Egypt has emerged as the dominant producer, accounting for about 94% of global production [1]. In 2020, the global fisheries and aquaculture production of mullet amounted to 291.2 thousand tons (TT), accounting for 3.5% of the total output. This figure increased to 374,072 TT in 2021 [2, 3]. Mullets are the most significant fish species in Egypt after tilapias, and their share amounts to around 18%, of the aggregate production, with more than 87% of this share coming from aquaculture [1]. The two primary species of mullet raised in Egypt are the thinlip grey mullet (*Chelon ramada*) and the flathead grey mullet (*Mugil cephalus*). These species are commonly grown using the polyculture method with tilapia in freshwater environments [4, 5]. Nevertheless, the aquaculture of these fish has faced difficulties in numerous countries due to the scarcity of freshwater in recent years.

Several potential solutions have been proposed to alleviate the adverse effects of freshwater scarcity on the growth of aquaculture. These include implementing water purification methods, integrating aquaculture with agriculture systems, and utilizing recirculating aquaculture systems [6, 7]. Furthermore, a novel trend has emerged in this particular situation, involving the utilization of GW (saline and brackish) for the purpose of establishing mariculture in arid regions [8, 9]. Egypt has large amounts of GW resources (the expected aggregate reserve will reach about 1,200 billion m^3^), making it the second largest source after the Nile River [10], and hence it plays a crucial role in economic elaboration and sustainable development across the country [11]. Saline and brackish GW which is unsuitable for use by humans, animals and for agricultural purposes, represents the majority of these aquifer systems [10, 12], making it a convenient water source for rearing euryhaline fish such as grey mullet species.

The main component of the cultured mullet species is *C. ramada*. This is because there is a large supply of fingerlings as bycatch, and it has a faster growth rate compared to all Mediterranean mullet species, save for *M. cephalus*, which now has very few juveniles [13]. However, *C. ramada* is expected to suffer from malnutrition and increased stress in such GW, which differs greatly from seawater in its characteristics and hardness with a high probability of the presence of contaminants such as heavy metals (HM) and pathogens. In this regard, contaminants such as HM can be concentrated in fish organs such as livers, gills and kidneys, even if they are present at very low concentrations in farm water, and thus the liver has been proposed as a biomarker for HM contamination [14–16]. In our earlier studies [8, 9] when GW was employed to develop desert mariculture for Florida red tilapia, a biofloc system was applied to enhance water quality and minimize the existence of pathogenic bacteria, thus overcoming water quality issues affecting fish performance and well-being. Moreover, probiotics were employed as water treatment to address issues associated with poor water quality and mitigate HM concentrations in both culture water and fish organs, when GW was used in farming European seabass (our data under publication). In this context, numerous natural substances have been recently employed as a substitute for antibiotics and chemical treatments in aquaculture techniques [17–20]. This is done to mitigate the negative impact of abiotic factors on cultivated fish, thereby enhancing their immunity and production, and potentially decreasing the occurrence of diseases [6, 21]. Medicinal plant derivatives, extracts, and essential oils have gained attention as effective and cost-efficient bioactive alternatives derived from biological sources. These alternatives are favored due to their high efficacy, safety and minimal environmental impact [17, 22–24].

Rosemary oil (RO) is an extract of the medicinal plant rosemary belonging to the Labiatae family. RO is abundant in antioxidants and retains functional extracts primarily comprised of 1,8-cineole, camphene, camphor, carnosic acid, carnosol, rosmarinic acid, and β- and α pinene [25]. Earlier investigations demonstrated that inclusion of rosemary extract improved the growth indices of African catfish (*Clarias gariepinus*) [26], the weight gain of the Nile tilapia (*Oreochromis niloticus*) [27], and the growth indices, innate immunity, and antioxidants of common carp (*Cyprinus carpio*) in response to crowding stress [28]. Furthermore, adding rosemary extract to tilapia subjected to aflatoxin B1 improved their performance [29]. Additionally, including RO with/without mixture of amylase and lipase enzymes in the *C. Ramada* diet reduced NH_3_ levels in the rearing water and promoted growth, digestive enzyme activity and intestinal health [1]. Zymogen Forte (ZF) is a pharmaceutical drug that promotes digestibility and treats indigestion resulting from dysfunction of the stomach, pancreas, intestines, or lack of bile secretion, and works as an appetite stimulant and anti-flatulence agent (https://gardeniapharmacies.com/product/zymogen-forte-tab/?lang=en). To our knowledge, no previous studies have been launched to explore the potential independent effect of RO or synergistic effect of RO + ZF on the performance, physiological alterations, and overall health in mullets, especially *C. ramada*. The objective of this study is to examine the potential improvement from adding RO and RO + ZF to the diet of *C. ramada* fish cultured in GW and to uncover the likely impacts on culture water, fish growth, and related hormones (growth hormone and insulin-like growth factor 1), antioxidant capacity, immune function, serum heat shock proteins 70 and 90 (involved in rebuilding damaged proteins in the body’s cells), and internal organ health.

## Materials and methods

### Location and water source criteria

This experimental study was conducted at El-Max Research Station for Applied Research (MRSAR), NIOF, Alexandria, Egypt. GW with a salinity of 18.5–19.5 ppt from 50 m depth was used to culture fish. Chemical analysis of GW showed: salinity 19.2 ± 0.1 ppt, pH 8.06 ± 0.1, total ammonia nitrogen (TAN) 0.41 ± 0.035 mg/L, manganese 85.2 ± 1.08 µg/L, iron 99.3 ± 2.2 µg/L, copper 5.3 ± 0.005 µg/ L, zinc 6.5 ± 0.002 µg/ L, cadmium 40.0 ± 1.0 µg/ L, chromium 66.0 ± 2.0 µg/ L, cobalt 50.0 ± 2.0 µg/ L, nickel 70.0 ± 5.0 µg/ L, lead 28.0 ± 3.0 µg/ L, and total hardness 3563.7 ± 2.73 mg/L.

### Diet preparation

The basal diets were developed by grinding and mixing feedstuffs (including ZF tablets) to be isonitrogenous (33.7% crude protein), and isolipidic (10.4% lipids). Afterward, fish oil, corn oil, RO, and 300 ml of water were combined and added to each kg of the mixture to form the pellets (Table 1). These pellets were then transformed into a dough with a diameter of 2 mm using laboratory pelletizing equipment (Tornado MG-200, Cairo, Egypt). The prepared diets were thereafter dried at room temperature and stored at 4 °C.

RO (almost 100% purity) was acquired from the local market (AREEJ Co., Cairo, Egypt, https://areejaromatherapy.com/product/rosemary-oil-15-ml/) and weighs 0.908 g per mL (density of RO is equal to 908 kg/m^3^; at 25 °C). ZF composition [Digestive enzymes combination with Antiflatulent agent: Amylase 5000 (NF units) + Protease 5000 (NF units) + Lipase 800 (NF units) + Ox-bile extract 20 mg + Hemicellulase (5000 I.U./gm) 40 mg + Pepsin (1:10.000 I.U.) 100 mg + Dimethyl polysiloxane (= Dimeticone) 20 mg + Vitamin B1 25 mg] was produced by ELNile Pharmaceuticals and Chemical Industries, Com, Egypt. The applied dose of RO is referenced by Yousefi et al. [28], while the dose of ZF is indicated by El-Dahhar [30], taking into account the economic cost of the diet.

**Table 1:** Feed formulation and chemical analysis of the basal diets containing different levels of rosemary oil and/or Zymogen Forte used to feed *Chelon ramada* for a 75-day rearing trial in brackish groundwater

Ingredients (g/kg)	Treatments^1^				
	T1	T2	T3	T4	T5
Fish meal (65% - anchovy)	60	60	60	60	60
Wheat (10 CP)	50	50	50	50	50
Poultry offal meal	100	100	100	100	100
Soybean (45-solv VN)	262	262	262	262	262
Gluten (corn)	90	90	89	89	89
Wheat bran	320	320	320	320	320
Fish oil	30	30	30	30	30
Corn oil	30	29.5	29.5	30	29
Yeast (Brewers)	30	30	30	30	30
Trace mineral premix^2^	3	3	3	3	3
Vitamin premix^2^	3	3	3	3	3
Gelatin	10	10	10	10	10
Dicalcium Phosphate	10	10	10	10	10
Vitamin C	1	1	1	1	1
Probiotic	1	1	1	1	1
Rosemary oil^**3**^	0	0.5	0.5	1	1
Zymogen Forte ^**4**^	0	0	1	0	1
Total	1000	1000	1000	1000	1000
**Chemical composition**					
DM, %	90.7766	90.7756	90.6827	90.7817	90.6817
CP, %	33.725	33.725	33.7519	33.6619	33.7519
Lipid, %	10.411	10.361	10.353	10.403	10.303
Fiber, %	4.2316	4.2316	4.2297	4.2297	4.2297
Ash, %	6.6071	6.6071	6.6054	6.6054	6.6054
Gross energy (GE), MJ/kg^5^	19.28197	19.26222	19.24022	19.25997	19.22047

^**1**^ Treatments: (T1) basal diet without rosemary oil or Zymogen Forte; (T2) basal diet with 0.5 g/kg feed rosemary oil; (T3) basal diet with 0.5 g/kg feed rosemary oil and 1 g/kg Zymogen Forte; (T4) basal diet with 1.0 g/kg feed rosemary oil; and (T5) basal diet with 1.0 g/kg feed rosemary oil and 1 g/kg Zymogen Forte

^**2**^ Vitamin and mineral premix: Premix: vitamin A (3300 IU), vitamin D3 (410 IU), vitamin B1 (133 mg), vitamin E(150 mg), vitamin B2 (580 mg), vitamin B6 (410 mg), vitamin B12 (50 mg), biotin (9330 mg), choline chloride (4000 mg), vitamin C (2660 mg), inositol (330 mg), para-amino benzoic acid (9330 mg), niacin (26.60 mg), pantothenic acid (2000 mg), manganese (325 mg), iron (200 mg), copper (25 mg), iodine, cobalt (5 mg)

^**3**^ Rosemary oil was purchased from AREEJ, Co. Cairo Egypt

^**4**^ Zymogen Forte 20 TAB (Digestive enzymes combination with Antiflatulent agent. Composition: Each tablet contains: Amylase 5000 (NF units) + Protease 5000 (NF units) + Lipase 800 (NF units) + Ox-bile extract 20 mg + Hemicellulase (5000 I.U./gm) 40 mg + Pepsin (1:10.000 I.U.) 100 mg + Dimethyl polysiloxane (= Dimeticone) 20 mg + Vitamin B1 25 mg.) Produced by ELNile For Pharmaceuticals and Chemical Industries, http://nilepharma.com.eg/en/home-ar/, Price: 26 EGP/20 tablets = 0.84 US$

Nitrogen free extract (NFE, %) = 100 - (crude protein + crude lipids + fibers + ash)

^**5**^ Gross energy was calculated based on protein, lipid, and carbohydrate values as 23.6, 39.5, and 17.2 KJ/g, respectively

### Experimental layout, fish and rearing facilities

A total of three hundred apparently healthy *C. ramada* juveniles were acquired from a commercial fish farm and relocated to MRSAR. For three weeks the fish were housed at a rate of 100 fish/tank in three concrete tanks (each 3 × 8 × 1.5 m, with 24 m^3^ water capacity) that were continuously aerated with an outlet air blower for acclimatization. During the acclimatization phase, the fish were given a commercial meal (30/5; protein/lipids) twice a day (09:00 and 14:00). Afterward, fish with similar initial weights (8.51 ± 0.01 g/fish) were assigned to experimental hapas for a 75-day growth trial.

This experiment utilized fifteen fiberglass tanks (each with 1500 L of water) filled with GW. Fish were evenly distributed at a density of 20 fish/tank, in the experimental tanks, with every three tanks representing one treatment. Throughout the trial, the tanks were consistently supplied with air using an air blower for a duration of 3 h. The fish were maintained under a natural light cycle, with 12 h of light followed by 12 h of darkness. Down-siphoning was performed every three days to clear uneaten feed and feces from the experimental tanks, with a daily 10% water exchange. Five treatments administered in triplicate were: T1: fish-fed BD without additives (control group); T2: fish-fed BD + 0.5 g RO / kg diet; T3: fish-fed BD + 0.5 g RO and 1 g ZF /kg diet; T4: fish-fed BD + 1 g RO /kg diet; T5: fish-fed BD + 1 g RO and 1 g ZF /kg diet. The fish were provided with experimental diets at a feeding rate equivalent to 3% of their body weight.

### Water quality assessment

#### Physicochemical parameters of water culture

During the study, the SensoDirect 150, a multiparameter portable photometer, was used to estimate water temperature (°C), pH, salinity (mS/cm converted to ppt), and dissolved oxygen (O2), daily on-site at approximately the same time (10:00 a.m.). TAN, unionized ammonia (NH_3_), nitrite (NO_2_), alkalinity, and hardness were weekly measured throughout the trial period, according to APAH [31]. A total of 1 L of water was taken from each tank at a depth of 0.4 m below the water’s surface. The portable photometer Hanna HI-96,715 Medium Range Ammonia was utilized to measure TAN. NH_3_ was calculated from TAN (mg/L), salinity, pH, and temperature data. The titrimetric method was used to calculate alkalinity [32]. Total hardness was assayed using the YSI Professional Plus Multiparameter Instrument.

#### Sampling, counting and identifying the main pathogenic bacterial groups in culture water

After the trial was concluded, water samples were obtained to measure the presence of harmful bacteria in the experimental water tanks using the previously stated protocols by PHE [33]. Water sample bottles were kept in the dark at a stable low temperature. Samples of water were collected in sterile 500 ml screw-cap bottles, as recommended by Austin [34]. For cultivation, 1 ml of each sample was diluted in 99 ml of saline and cultured using the pour plate method on a nutrient agar plating medium at 30 °C for 18–24 h. The enumeration of microorganisms tested was represented as CFU/100 mL. Bacterial testing was conducted using membrane filtration methods defined in ISO 9308/1 (2000) and 7899/2 (2000). Diluted samples (1 ml) were filtered via a 0.45 μm pore (47 mm diameter) sterile cellulose membrane filter with grid. *Faecal streptococcus* membranes were placed on Slanetz and Bartley media after 72 h of incubation at 37 °C. The dark red colonies were counted to identify *Vibrio sp*. The membranes were placed on thiosulphate citrate bile salt sucrose (TCBS) agar and incubated at 37 °C for 24 h. Large green and/or yellow colonies were considered to be *Vibrio sp*. The numbers of *Salmonella species* were identified and counted on bismuth sulfite agar and *Salmonella-Shigella (SS)* agar media, and black colonies were counted after 18–24 h of incubation at 35–37 °C. *Aeromonas sp.* numbers were identified using an *Aeromonas* isolation medium. After 18–24 h of incubation at 35–37 °C, dark green opaque colonies with dark cores were counted. To detect and count *Staphylococcus aureus*, membranes were placed on mannitol salt agar and incubated at 35 °C for 18–24 h, and then the yellow colonies were counted. To detect and count *Pseudomonas sp*. membranes were put on *Pseudomonas* isolation agar. Blue-green colonies appeared after 40–48 h of incubation at 35 °C. The following media were used in this work, and their composition is expressed in g/L. The pH of the media was adjusted to 7.5 before sterilization, and the autoclave was running for 15 min at 121 °C Celsius.

A) Nutrient agar medium [35] composed of: yeast extract, 2; beef extract, 1; peptone, 5; sodium chloride, 5. Agar (15–20) was added for obtaining nutrient agar medium.
B) Slanetz and Bartley media [36] composed of tryptose 20, yeast extract 5, dextrose 2, dipotassium phosphate 4, sodium azide 0.4, agar 10, 2,3,5 triphenyl tetrazolium chloride 0.1. The final pH was 7.2 ± 0.2 at 25 °C.
C) Mannitol salt agar [37] used for isolating *Staphylococcus spp*. Peptone complex, 10; beef extract, 1; sodium chloride, 75; mannitol 10; phenol red, 0.025 and agar 15.
D) Thiosulfate citrate bile salt sucrose agar (TCBS) [38] used for isolating *Vibrio spp*. Yeast extract, 5; peptone, 10; sodium thiosulfate, 10; sod-citrate, 10; Ox bile, 8; sucrose, 20; sodium chloride, 10; ferric chloride, 1; Bromothymol blue, 0.04; thymol blue, 0.04 and agar, 14.
E) Aeromonas agar [35] for the isolation of *Aeromonas sp*. which is composed of: proteose peptone; 5.0, yeast extract; 3.0, l. lysine monohydrochloride; 3.5, l. arginine monohydrochloride; 2.0, sorbitol; 3.0, inositol; 2.5, lactose; 1.5, xylose; 3.75, bile salts #3; 3.0, sodium thiosulfate; 10.67, sodium chloride; 5.0, ferric ammonium citrate; 0.8, bromothymol blue; 0.04, thymol blue; 0.04 and agar; 12.5.
F) *Salmonella Shigella* Agar [35] used to isolate *Salmonella* and *Shigella.* It is composed of: beef extract; 5.0, polypeptone; 5.0, lactose; 10.0, Sodium citrate; 8.5, Ferric Citrate 10.0, Sodium thiosulfate; 8.5, bile salts #3; 8.5, Neutral Red; 0.025, agar; 13.5 and Brilliant green, 0.330 mg.

### Growth indices and feed utilization calculation

Prior to the final sampling, a mandatory 24-hour fasting interval was enforced for each experimental group. At the conclusion of the rearing trial, growth and feed utilization indices were calculated using the following equations: [39, 40].

$$WG,\,g = F{W_{75}} - I{W_0}$$

$$Average\,daily\,gain,\,\left( {ADG,\,{g \mathord{\left/{\vphantom {g {day}}} \right.\kern-\nulldelimiterspace} {day}}} \right) = \frac{{WG,\,g}}{{75}}$$

$${{SGR\,\% } \mathord{\left/{\vphantom {{SGR\,\% } {day = \frac{{Ln\,F{W_{75}} - Ln\,I{W_0}}}{{75}}}}} \right.\kern-\nulldelimiterspace} {day = \frac{{Ln\,F{W_{75}} - Ln\,I{W_0}}}{{75}}}} \times 100$$

$$Feed\,\operatorname{int} ake\left( {FI,\,g\,Fis{h^{ - 1}}\,da{y^{ - 1}}} \right) = \frac{{Feed\,consumption}}{{Avereage\,biomass,\,g \times 75}}$$

$$FCR = \frac{{FI,\,g}}{{WG,\,g}}$$

$$SR\,\% = \frac{{{N_{75}}}}{{{N_0}}} \times 100$$

Where:

WG: Weight gain; FW_75_: Final weight; IW_0_: Initial weight; SGR: Specific growth rate; FCR: Feed conversion ratio; SR: Survival rate; N_70_: Final number; N_0_: Initial number.

### Blood sampling and analyses

#### Complete blood count (CBC)

At the completion of the experiment, blood samples were obtained from four fish from each tank (*n* = 12 per treatment) following a 24-hour fasting period. The fish were sedated using 50 mg clove oil/L [17, 41]. A complete CBC was performed by drawing blood from the caudal vein using 3.0-mL heparinized syringes. Hemoglobin levels were determined employing the cyanomet hemoglobin method using Drabkin’s solution [42]. White blood cell (WBC; 10^3^ mm^− 3^) counts were performed using a standard Neubauer hemocytometer chamber with Shaw’s solution as the diluent. Furthermore, differential leukocyte counts were performed by light microscopy (Optika, Via Rigla, Ponteranica, Italy) using Giemsa-stained smears. Hematocrit (Ht; %) was assessed by filling capillary hematocrit tubes that were centrifuged (8400 ˣ g, 10 min) using a hematocrit microcentrifuge (Krebs, Bunsen, EU). The hematocrit concentrations were documented using a centrifuge combo-reader. The microhematocrit technique was employed to assess the packed cell volume (PCV) and mean corpuscular volume (MCV) values [43].

#### Serum analyses

Three milliliters of blood were preserved in non-heparinized tubes, then centrifuged at 3000 × g for 15 min at 4 °C. Serum total protein (TP) and albumin (ALB) were assessed using the procedures of Doumas et al. [44] and [45], respectively. Globulin (GLO) concentration was computed mathematically. Immunoglobulin (IgM) was estimated by precipitating Ig with polyethylene glycol and terminating the initial and final TP [46]. Serum creatinine (CRE) was determined according to Heinegård and Tiderström [47]. Serum cortisol (COR) was assessed using the methods described by Sadoul and Geffroy [48] and serum glucose (GLU) was evaluated following the method of P Trinder [49]. Plasma levels of complement 3 (C3) and complement 4 (C4) were assayed following the manufacturer’s protocol using ELISA kits (Zhejiang, China) [50].

#### Antioxidants status

The activities of catalase (CAT) and superoxide dismutase (SOD) were determined using ZellBio’s kits (GmbH, Germany) as described by Yousefi et al. [51] and Hoseini and Yousefi [52]. Serum malondialdehyde (MDA) was determined using the Uchiyama and Mihara method [53]. The total antioxidant capacity (TAC) was measured (Hitachi 7600D, Hitachi, Tokyo, Japan) following the protocol of Koracevic et al. [54].

### Enzyme-linked Immunosorbent Assay (ELISA)

The enzyme-linked Immunosorbent Assay (ELISA) is a serological test that uses a mixture of distinct antibodies along with enzymes to detect the presence of antigens. The antigen binding to an antibody can be confirmed by adding a substrate that will be hydrolyzed by the marker enzyme and can be detected immediately by color changing using a spectrophotometer [55].

#### *Heat shock proteins* 70 *and 90 (HSP70* & *HSP90)*

Samples of plasma were collected from anesthetized fish (*n* = 12/treatment group) and centrifuged (Sorvall ST 16R, Thermo Fisher Scientific, Waltham, Massachusetts), then immediately aliquoted and stored at − 80 ^0^C. The methodological protocol was detailed by Breuninger et al. [56] and Pawlik-Gwozdecka et al. [57].

#### Growth hormone (GH) and insulin-like growth factor 1 (*IGF1)*

The quantification of GH in the fish plasma was conducted according to the manufacturer’s instructions using fish-specific GH kits (https://www.cusabio.com/uploadfile/Ins/CSB-E12121Fh.pdf). Furthermore, the measurement of IGF 1 levels in fish plasma was conducted using fish-specific IGF1 kits (https://www.cusabio.com/uploadfile/Ins/CSB-E12122Fh.pdf).

### Histomorphology changes in liver and middle intestine

The liver and intestine segments (mid-section) of three fish (*n* = 9 per treatment) were collected and immersed in a 4% paraformaldehyde solution for 24 h. Subsequently, the samples were transferred to a series of ethanol concentrations to gradually remove moisture from the fixed samples. After a 24-hour duration, the samples were immersed in a solution containing 70% ethanol. After undergoing drying and cleaning operations, the tissue samples were embedded in paraffin and then sectioned to a thickness of 5 μm using a rotary microtome (RM 2035, Leica Microsystems, Wetzlar, Germany). The serial sections were stained using the hematoxylin and eosin (H&E) method [58]. The histomorphometric photomicrographs were acquired using a Leica EC3 digital camera connected to a Leica DM500 microscope.

### Statistical analysis

Means and standard errors of the mean (SEM) were calculated using SPSS Software version 26 through one-way and two-way analysis of variance (ANOVA) and Duncan’s test. The treatments (T1, T2, T3, T4, and T5) were subjected to analysis using a one-way ANOVA. After excluding T1 (control), the remaining treatments (T2, T3, T4, and T5) were analyzed using two-way ANOVA to determine the interaction effect of RO and ZF. A normality test was carried out using the Shapiro-Wilk test. The significance level was set at *P <* 0.05.

## Results

### Water quality criteria

#### Physicochemical parameters of water in culture tanks

**Fig. 1:**
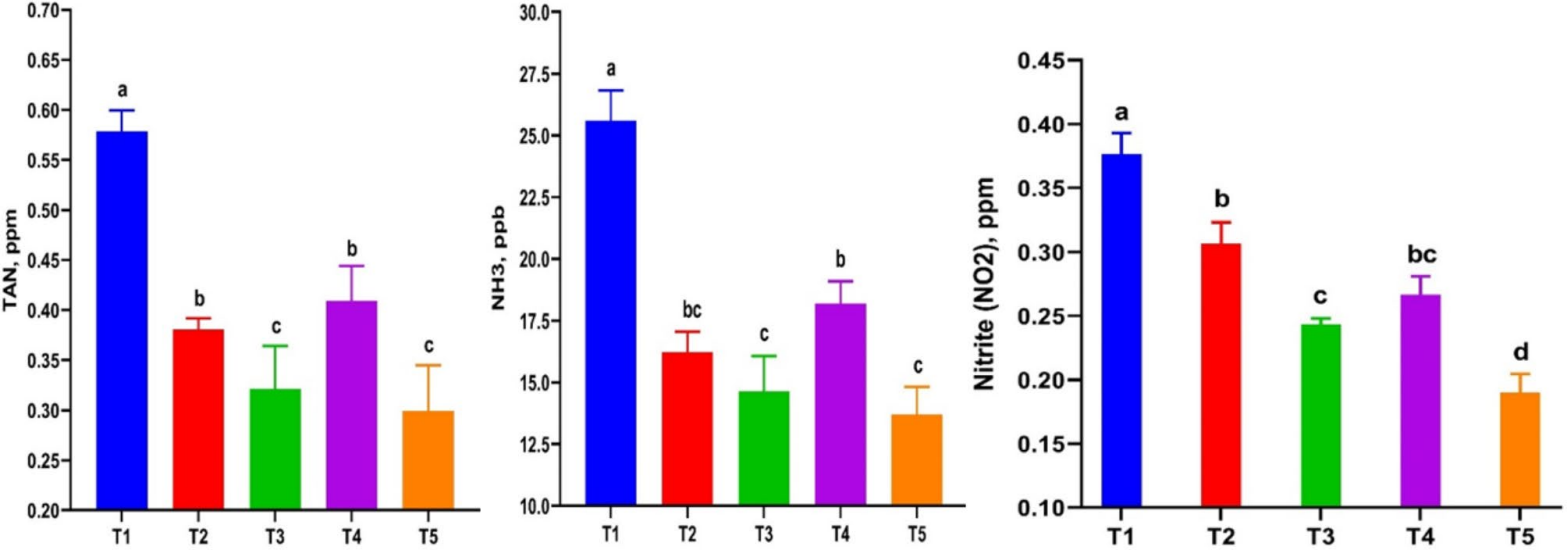
Total ammonia nitrogen (TAN), unionized ammonia (NH_3_) and nitrite (NO_2_) concentrations in culture water tanks of *Chelon ramada* fed a basal diet containing varying levels of rosemary oil and/or Zymogen Forte during a 75-day rearing trial using brackish groundwater. (T_1_) = fish fed BD without RO or ZF (control); (T_2_) = fish fed the BD containing 0.5 g of RO kg^− 1^; (T_3_) = fish fed BD containing 0.5 g of RO and 1 g of ZF kg^− 1^; (T_4_) = fish fed BD containing 1.0 g of RO kg^− 1^; and (T_5_) = fish fed BD containing 1 g of RO and 1 g of ZF kg^− 1^. Abbreviations: BD = basal diet; RO = rosemary oil; ZF = Zymogen Forte

No major fluctuations (*P < 0.05*) occurred between the experimental groups in relation to the following parameters: water temperature 27.50 °C (25.66–28.43), pH 7.63 (7.21–7.87), dissolved oxygen 6.58 (6.13–6.87) mg/L, salinity 19.2 (18.5–19.5) ppt, alkalinity 212.2 (191.9-225.4) mg/L, and hardness 3895.3 (3689–4013) mg/L. Significant changes (*P < 0.05*) were detected in the contents of nitrogen by-products (TAN, NH_3_, and NO_2_) between treatments, with the greatest decreases in TAN, NH_3_, and NO_2_ values ​​ observed in T3 and T5 (Fig. 1). The four treated groups showed a decrease (*P < 0.05*) in these factors compared to the control group, with the combined effect of RO and ZF in T3 and T5 leading to the most desirable decreases. Increasing the RO dose from 0.5 g/kg in T2 to 1 g/kg in T4 did not improve the removal of these compounds from the rearing water.

#### Pathological bacterial load in culture tank water

**Fig. 2:**
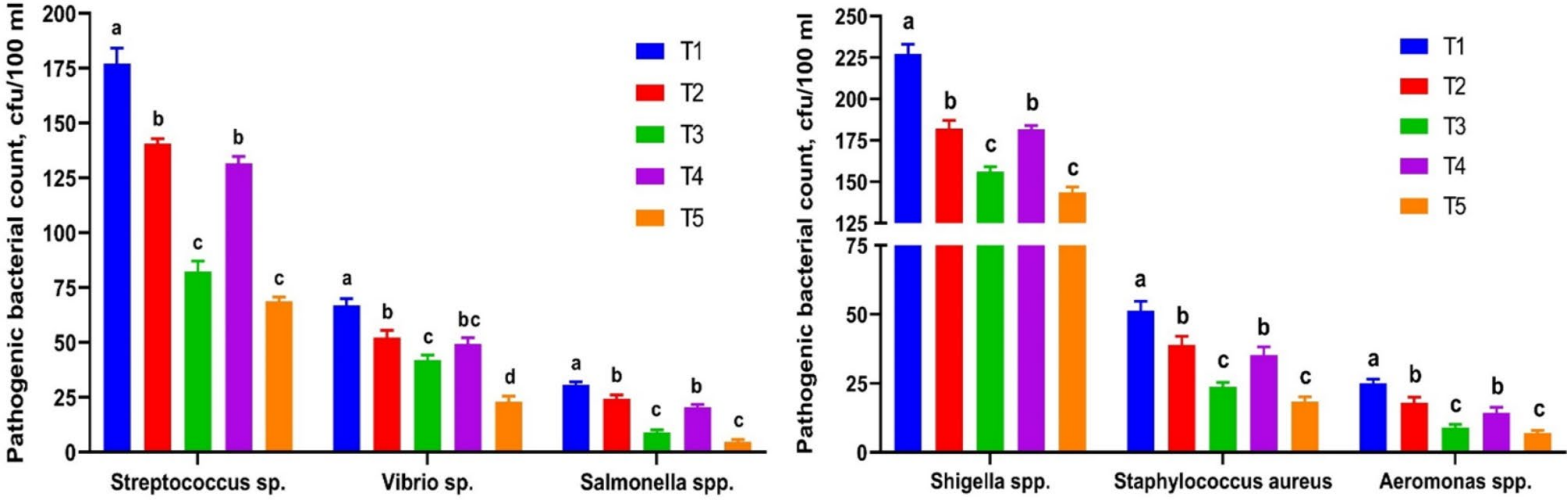
Pathogenic bacterial load (CFU/100 ml) numbers in culture water tanks of *Chelon ramada* fed a basal diet containing varying levels of rosemary oil and/or Zymogen Forte for a 75-day rearing trial using groundwater. (T_1_) = fish fed BD without RO or ZF “control”; (T_2_) = fish fed the BD containing 0.5 g of RO kg^− 1^; (T_3_) = fish fed BD containing 0.5 g of RO and 1 g of ZF kg^− 1^; (T_4_) = fish fed BD containing 1.0 g of RO kg^− 1^; and (T_5_) = fish fed BD containing 1 g of RO and 1 g of ZF kg^− 1^; BD = basal diet, RO = Rosmery oil; ZF = Zymogen Forte

Dietary inclusion of RO and RO + ZF significantly (*P < 0.05*) affected the presence of pathogenic bacteria in culture water of *C. ramada* (Fig. 2). An overall decrease in the number of pathogenic bacteria (CFU/100 mL) in *C. ramada* growing water was noted in all supplemented groups compared to the control. The integrated effect of ZF and RO at low (0.5 g/kg) and high (1 g/kg) doses led to the most substantial (*P < 0.05*) decline in the numbers of *Streptococcus* spp., *Salmonella* spp., *Shigella* spp., *Staphylococcus aureus*, and *Aeromonas* spp. The complementary influence of ZF and RO at the high dose resulted in the most desirable reduction in the numbers of *Vibrio* spp. in T5. Moreover, the interaction between ZF and RO at the low dose (0.5 g/kg) in T_3_ maintained the same statistical values ​​as T4. Increasing the RO dose from 0.5 g/kg to 1 g/kg did not lead to a major reduction in the number of pathogenic bacteria measured in T4 compared to T2.

### Growth performances

The greatest increase in growth indices (FW, WG, ADG, and SGR) and the best numerical FCR were reported for T5, where the interaction between RO (1 g/kg) and ZF occurred. Overall, the inclusion of RO and/or ZF in the diet of *C. ramada* notably (*P < 0.05*) increased growth and FCR compared to fish fed the control diet (Table 2). Although T5 showed the best numerical FCR value, no significant differences (*P > 0.05*) were reported among the treated groups (T2, T3, T4, and T5). Fish raised in T3 were affected by the interaction between RO (0.5 g/kg) and ZF and showed better growth indicators than those raised in T2 and T4 and fed RO at 0.5 and 1 g/kg, respectively. Moreover, increasing the RO level to 1 g/kg (T4) resulted in better growth performance compared to fish fed RO at 0.5 g/kg (T2). Survival rates were higher in all enriched groups compared to the control group (T1), with the highest values ​​observed in T3 and T5.

**Table 2:** Growth performance and feed utilization of *Chelon ramada* fed a basal diet supplemented with varing levels of rosemary oil and/or Zymogen Forte during a 75-day rearing trial in brackish groundwater

parameters	Treatments					*P*-Value		
	T1	T2	T3	T4	T5	RO	ZF	RO*ZF
IW (g/fish)	8.50 ± 0.02	8.50 ± 0.02	8.52 ± 0.02	8.52 ± 0.01	8.51 ± 0.01	-	-	-
FW (g/fish)	21.30 ± 0.31^**e**^	22.87 ± 0.35^**d**^	28.21 ± 0.35^**b**^	26.47 ± 0.32^**c**^	30.90 ± 0.77^**a**^	0.000	0.000	0.377
WG (g/fish)	12.80 ± 0.32^**e**^	14.36 ± 0.37^**d**^	19.70 ± 0.36^**b**^	17.95 ± 0.31^**c**^	22.40 ± 0.78^**a**^	0.000	0.000	0.393
ADG(g/fish/day)	0.171 ± 0.004^**e**^	0.192 ± 0.005^**d**^	0.263 ± 0.005^**b**^	0.239 ± 0.004^**c**^	0.299 ± 0.010^**a**^	0.000	0.000	0.396
SGR (%/fish/day)	1.22 ± 0.02^**e**^	1.32 ± 0.02^**d**^	1.60 ± 0.02^**b**^	1.51 ± 0.02^**c**^	1.72 ± 0.03^**a**^	0.000	0.000	0.183
Survival (%)	76.67 ± 3.33^**d**^	86.33 ± 1.67^**c**^	98.33 ± 1.67^**a**^	92.67 ± 1.67^**b**^	96.67 ± 3.33^**a**^	0.029	0.000	0.009
Feed Intake, g/fish	28.07 ± 2.47^**bc**^	25.02 ± 0.59^**c**^	31.85 ± 0.36^**ab**^	31.13 ± 0.61^**b**^	35.61 ± 1.11^**a**^	0.000	0.000	0.143
FCR	2.20 ± 0.25^**a**^	1.74 ± 0.01^**b**^	1.62 ± 0.02^**b**^	1.73 ± 0.01^**b**^	1.59 ± 0.01^**b**^	0.147	0.000	0.608

Data are presented as mean ± SEM of 3 replicates, with different letters in the same row presenting significant differences (*p* < 0.05). (T_1_) = fish fed BD without RO or ZF (control); (T_2_) = fish fed the BD containing 0.5 g of RO kg^− 1^; (T_3_) = fish fed BD containing 0.5 g of RO and 1 g of ZF kg^− 1^; (T_4_) = fish fed BD containing 1.0 g of RO kg^− 1^; and (T_5_) = fish fed BD containing 1 g of RO and 1 g of ZF kg^− 1^; BD = basal diet, RO = rosemary oil; ZF = Zymogen Forte; RO * ZF = interaction; IW = initial weight; FW = final weight; WG = weight gain; ADG = average daily gain; SGR = specific growth rate; FCR = feed conversion ratio

### Blood hematobiochemistry indices

#### CBC

Hemoglobin (Hb), red blood cells (RBCs), hematocrit, MCV, MCH, MCHC, and lymphocytes were significantly (*P* < 0.05) higher in fish from the supplemented groups compared to those in the control group (Table 3). The higher RO dose significantly (*P* < 0.05) improved white blood cell (WBC) counts in T4 compared to the control group, an effect not observed with the lower RO dose in T2. The combined effect of ZF and RO (both low and high doses) in groups T3 and T5 resulted in increased (*P* < 0.05) in the levels of Hb, RBCs, hematocrit, and WBCs compared with other treatments. Moreover, the highest leukocytes (WBCs) numbers were in group T5, the greatest neutrophils numbers were found in group T2, while groups T3 and T4 showed the highest lymphocyte counts.

**Table 3:** Complete blood count (CBC) of *Chelon ramada* fed a basal diet supplemented with varing levels of rosemary oil and/or Zymogen Forte during a 75-day rearing trial in brackish groundwater

CBC	Treatments					*P*-Value		
	T1	T2	T3	T4	T5	RO	ZF	RO*ZF
Hb (g/100 ml)	10.15 ± 0.09^**c**^	11.35 ± 0.20^**b**^	11.60 ± 0.06^**ab**^	11.50 ± 0.12^**b**^	11.90 ± 0.06^**a**^	0.106	0.030	0.560
RBCs (×10^6^/µL)	3.24 ± 0.02^**c**^	3.49 ± 0.02^**b**^	3.60 ± 0.01^**a**^	3.45 ± 0.03^**b**^	3.59 ± 0.03^**a**^	0.358	0.001	0.602
Hematocrit(HCT%)	29.21 ± 0.13^**c**^	32.03 ± 0.23^**b**^	33.49 ± 0.06^**a**^	32.05 ± 0.23^**b**^	32.91 ± 0.30^**a**^	0.248	0.001	0.215
MCV (µm3/cell)	90.15 ± 0.09^**c**^	91.90 ± 0.12^**b**^	93.15 ± 0.20^**a**^	92.9 ± 0.12^**a**^	91.80 ± 0.17^**b**^	0.295	0.644	0.000
MCH (pg/cell)	28.40 ± 0.23^**b**^	31.15 ± 0.20^**a**^	31.65 ± 0.20^**a**^	31.40 ± 0.69^**a**^	31.80 ± 0.12^**a**^	0.612	0.269	0.898
MCHC (%)	31.50 ± 0.29^**b**^	33.90 ± 0.18^**a**^	33.98 ± 0.14^**a**^	33.80 ± 0.70^**a**^	34.64 ± 0.19^**a**^	0.279	0.261	0.348
WBCs (×10^3^/µL)	81.85 ± 0.14^**d**^	84.85 ± 0.66^**d**^	97.00 ± 0.92^**b**^	89.00 ± 1.15^**c**^	105.40 ± 2.08^**a**^	0.001	0.000	0.146
Lymphocyte (%)	68.00 ± 0.15^**c**^	79.00 ± 0.58^**b**^	86.50 ± 0.87^**a**^	86.50 ± 0.87^**a**^	77.50 ± 0.87^**b**^	0.001	0.001	0.000
Neutrophils (%)	14.0 ± 0.58^**b**^	27.5 ± 1.73^**a**^	8.50 ± 0.87^**c**^	8.0 ± 0.58^**c**^	15.5 ± 2.60^**b**^	0.007	0.010	0.000
Monocyte (%)	4.5 ± 0.29	3.5 ± 0.29	3.5 ± 0.29	3.5 ± 0.29	5.0 ± 1.15	0.267	0.267	0.267
Eosinophil (%)	2.5 ± 0.29	1.5 ± 0.29	1.5 ± 0.29	2.0 ± 0.00	2.0 ± 0.58	0.195	1.000	1.000

Data are presented as mean ± SEM of 3 replicates, with different letters in the same row presenting significant differences (*p* < 0.05). (T_1_) = fish fed BD without RO or ZF (control); (T_2_) = fish fed the BD containing 0.5 g of RO kg^− 1^; (T_3_) = fish fed BD containing 0.5 g of RO and 1 g of ZF kg^− 1^; (T_4_) = fish fed BD containing 1.0 g of RO kg^− 1^; and (T_5_) = fish fed BD containing 1 g of RO and 1 g of ZF kg^− 1^; BD = basal diet, RO = rosemary oil; ZF = Zymogen Forte, RO*ZF = Interaction

#### Non-specific humoral immune factors

**Fig. 3:**
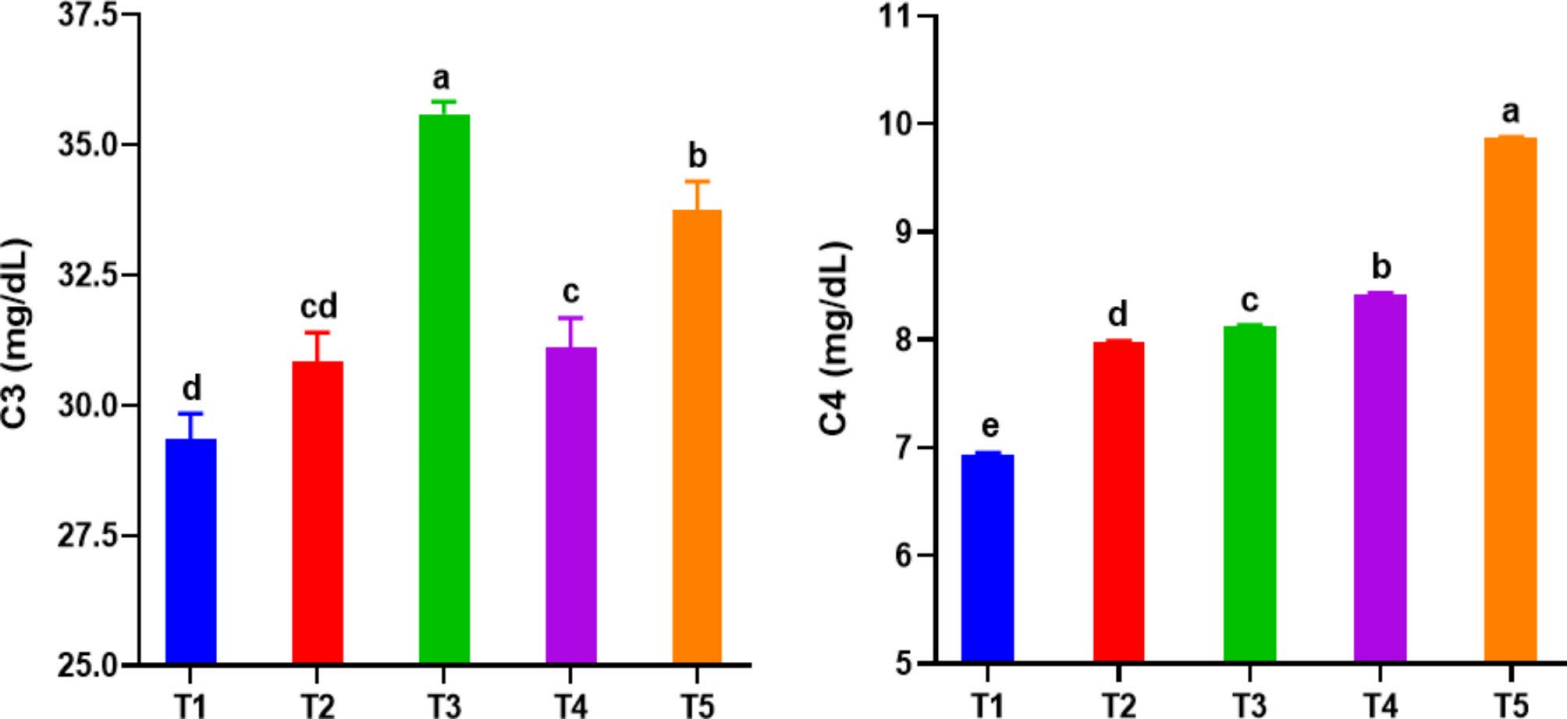
Complement component 3 (C3) and complement component 4 (C4) in *Chelon ramada* fed a basal diet containing varying levels of rosemary oil and/or Zymogen Forte for a 75-day rearing trial using brackish groundwater. Data are presented as mean ± SEM of 3 replicates, where (T_1_) = fish fed BD without RO or ZF “control”; (T_2_) = fish fed the BD containing 0.5 g of RO kg^− 1^; (T_3_) = fish fed BD containing 0.5 g of RO and 1 g of ZF kg^− 1^; (T_4_) = fish fed BD containing 1.0 g of RO kg^− 1^; and (T_5_) = fish fed BD containing 1 g of RO and 1 g of ZF kg^− 1^; BD = basal diet, RO = rosemary oil; ZF = Zymogen Forte

Table 4 shows improvements (*P < 0.05*) in the values ​​of immune indices (TP, ALB, GLU, and IgM) of *C. ramada* depending on the dose of RO and the interaction between RO and ZF. IgM and ALB levels increased significantly in all augmented groups compared to the control group, with the highest IgM values ​​in favor of T4 and ALB in favor of T5 and T2. The inclusion of high-dose RO at T4 and the interactive effect of RO and ZF (T3 and T4) resulted in an increase (*P < 0.05*) in serum TP compared to the control treatment, with no significant differences between these groups. No significant changes in GLO were observed between groups as a result of the addition of RO and RO + ZF. Significant improvements (*P < 0.05*) were observed in C3 and C4 in fish-fed diets treated with RO and RO + ZF compared to the control group (Fig. 3). Except for T2, all fortified groups showed considerable increases in C3 and C4 compared to the control group, with the highest values ​​favoring T3 and T5, respectively. The interaction effect of ZF and RO at low and high doses resulted in higher C3 and C4 values ​​being recorded in T3 and T5, respectively, while the high dose of RO at T4 significantly improved the C4 value compared to T3. The lowest values ​​of ALB, IgM, and C4 were observed in T1 (control group), while T1 and T2 shared the lowest C3 value.

**Table 4:** Serum immune indices of *Chelon ramada* fed a basal diet supplemented with varing levels of rosemary oil and/or Zymogen Forte during a 75-day rearing trial in brackish groundwater

Serum immune parameters	Treatments					*P*-Value		
	T1	T2	T3	T4	T5	RO	ZF	RO*ZF
TP(g/dL)	5.085 ± 0.113^**b**^	5.105 ± 0.147^**b**^	5.530 ± 0.144^**a**^	5.720 ± 0.087^**a**^	5.585 ± 0.113^**a**^	0.021	0.336	0.041
ALB(g/dL)	1.325 ± 0.072^**d**^	1.775 ± 0.009^**ab**^	1.595 ± 0.055^**c**^	1.655 ± 0.003^**b**^	1.905 ± 0.038^**a**^	0.022	0.327	0.000
GLO(g/dL)	3.760 ± 0.185^**ab**^	3.330 ± 0.139^**b**^	3.935 ± 0.199^**a**^	4.065 ± 0.089^**a**^	3.680 ± 0.150^**ab**^	0.114	0.585	0.008
ALB/GLO	0.378 ± 0.037^**b**^	0.539 ± 0.020^**a**^	0.413 ± 0.035^**b**^	0.410 ± 0.009^**b**^	0.525 ± 0.032^**a**^	0.700	0.878	0.002
IgM (mg/ml)	12.25 ± 0.087^**d**^	13.75 ± 0.029^**c**^	13.25 ± 0.028^**c**^	18.00 ± 0.404^**a**^	17.10 ± 0.462^**b**^	0.000	0.052	0.534

Data are presented as mean ± SEM of 3 replicates, with different letters in the same row presenting significant differences (*p* < 0.05). (T_1_) = fish fed BD without RO or ZF (control); (T_2_) = fish fed the BD containing 0.5 g of RO kg^− 1^; (T_3_) = fish fed BD containing 0.5 g of RO and 1 g of ZF kg^− 1^; (T_4_) = fish fed BD containing 1.0 g of RO kg^− 1^; and (T_5_) = fish fed BD containing 1 g of RO and 1 g of ZF kg^− 1^; BD = basal diet, RO = rosemary oil; ZF = Zymogen Forte, RO*ZF = Interaction; TP = total protein; ALB = albumin; GLO = globulin; IgM = immunoglobulin

#### Antioxidant status

**Fig. 4:**
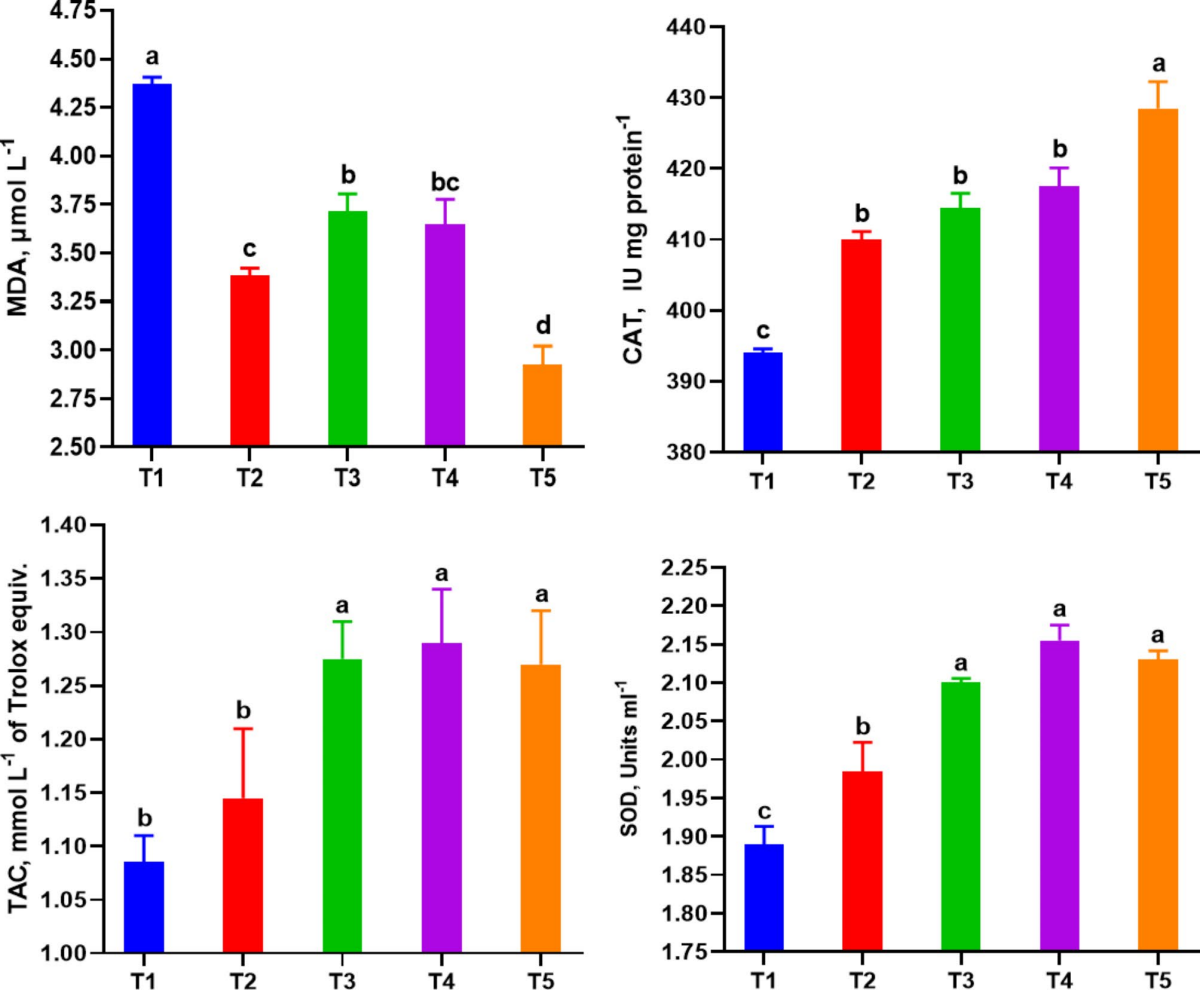
Plasma levels of antioxidant parameters (TAC, CAT, SOD and MDA) in *Chelon ramada* fed a basal diet containing varying levels of rosemary oil and/or Zymogen Forte for a 75-day rearing trial using groundwater. Data are presented as mean ± SEM of 3 replicates, where (T_1_) = fish fed BD without RO or ZF “control”; (T_2_) = fish fed the BD containing 0.5 g of RO kg^− 1^; (T_3_) = fish fed BD containing 0.5 g of RO and 1 g of ZF kg^− 1^; (T_4_) = fish fed BD containing 1.0 g of RO kg^− 1^; and (T_5_) = fish fed BD containing 1 g of RO and 1 g of ZF kg^− 1^; BD = basal diet, RO = rosemary oil; ZF = Zymogen Forte; TAC = total antioxidative capacity; CAT = catalase, SOD = superoxide dismutase; MDA = malondialdehyde

A general improvement in blood antioxidant activity has been reported in fish treated with RO and RO + ZF (Fig. 4). When compared to the control group (T1), all treated groups of fish exhibited a noteworthy reduction in MDA levels and a notable increase in CAT, TAC, and SOD, except for T2, which had the same TAC level as T1. The high dose of RO in T4 and the interaction between ZF and RO at low and high doses in T3 and T5, respectively resulted in higher TAC and SOD levels compared to the other groups. The combined effect of ZF and RO at the high dose (T5) led to the most desirable reduction in MDA levels and the greatest increase in CAT values in T5 group.

#### Stress markers (creatinine, cortisol and glucose)

**Fig. 5:**
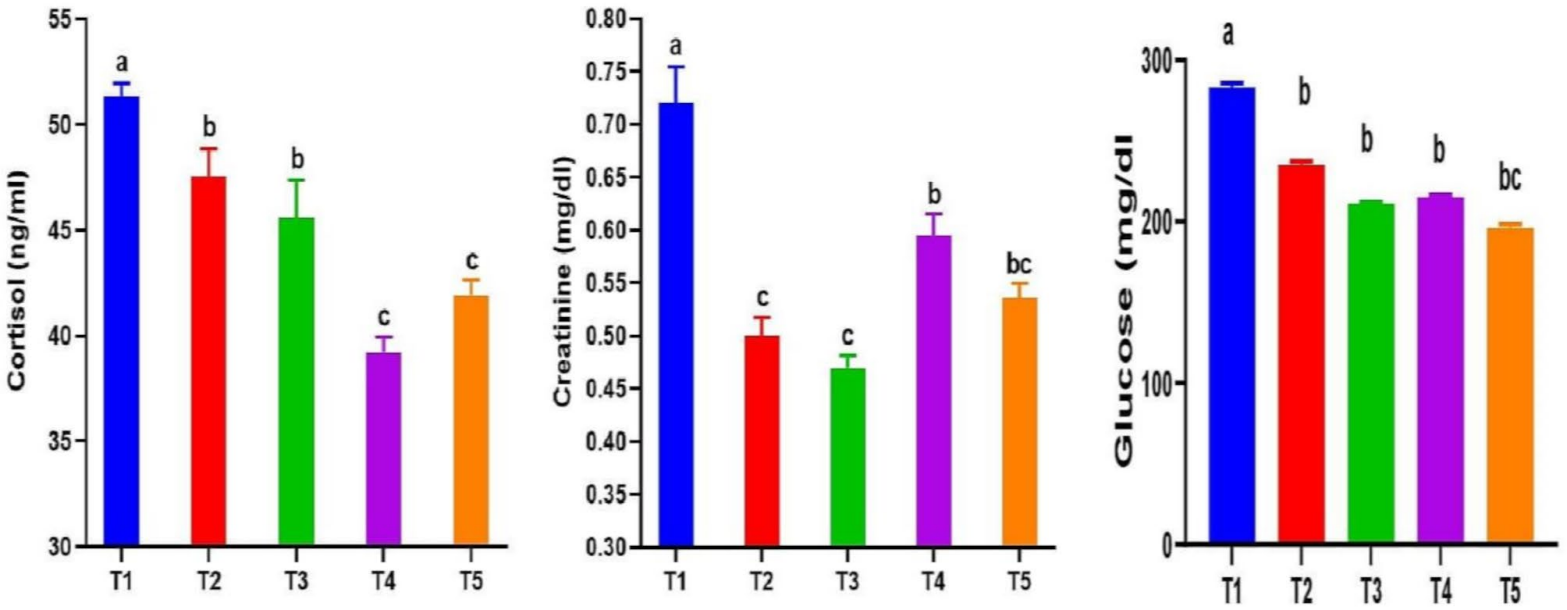
Serum stress markers (cortisol, creatinine and glucose) levels in *Chelon ramada* fed a basal diet containing varying levels of rosemary oil and/or Zymogen Forte for a 75-day rearing trial using brackish groundwater. Data are presented as mean ± SEM of 3 replicates, where (T_1_) = fish fed BD without RO or ZF “control”; (T_2_) = fish fed the BD containing 0.5 g of RO kg^− 1^; (T_3_) = fish fed BD containing 0.5 g of RO and 1 g of ZF kg^− 1^; (T_4_) = fish fed BD containing 1.0 g of RO kg^− 1^; and (T_5_) = fish fed BD containing 1 g of RO and 1 g of ZF kg^− 1^; BD = basal diet, RO = rosemary oil; ZF = Zymogen Forte

Statistically significant decreases (*P* < 0.05) in the concentrations of plasma stress markers (COR, CRE, and GLU) were noted in fish that were given diets treated with RO and RO + ZF, as compared to the control group (Fig. 5). Lower COR levels were reported for fish in all treatment groups, with the lowest values​​ favoring T4 and T5. Moreover, fish in all tested groups showed lower CRE values, ​​with the lowest levels recorded in T2, T3, and T5. In addition, fish fed RO and RO + ZF treated diets had lower levels of GLU than the control group, with the most desirable decrease favoring T5. Low-dose RO in T2 and T3 had a better impact in lowering CRE levels, while high-dose RO in T4 and T5 delivered a better effect in reducing COR levels. The interaction between ZF and RO at a low dose (0.5 g/kg) in T3 and a high dose (1 g/kg) in T5 did not show a better effect in lowering COR, CRE, and GLU levels.

#### Heat shock proteins

**Fig. 6:**
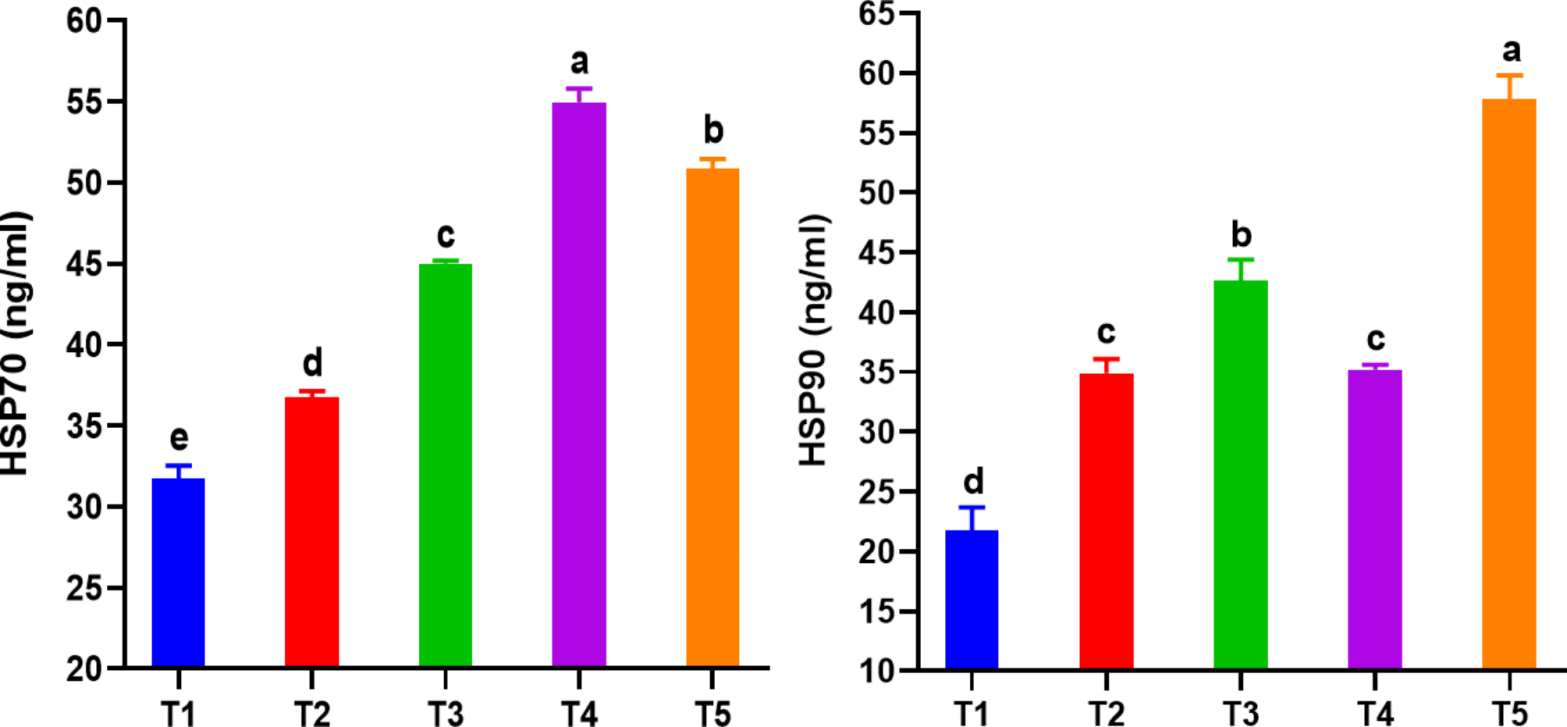
Serum heat shock proteins 70 and 90 in *Chelon ramada* fed a basal diet containing varying levels of rosemary oil and/or Zymogen Forte for a 75-day rearing trial using brackish groundwater. Data are presented as mean ± SEM of 3 replicates, where (T_1_) = fish fed BD without RO or ZF “control”; (T_2_) = fish fed the BD containing 0.5 g of RO kg^− 1^; (T_3_) = fish fed BD containing 0.5 g of RO and 1 g of ZF kg^− 1^; (T_4_) = fish fed BD containing 1.0 g of RO kg^− 1^; and (T_5_) = fish fed BD containing 1 g of RO and 1 g of ZF kg^− 1^; BD = basal diet, RO = rosemary oil; ZF = Zymogen Forte; HSP70 = heat shock protein 70; HSP90 = heat shock protein 90

HSP70 and HSP90 improved (*P < 0.05*) with the inclusion of RO and RO + ZF. These levels were consistently higher in all tested groups compared to the control group (Fig. 6). The significant interaction (*P < 0.05*) between RO and ZF was evident in improving HSP90 levels, scoring the highest values ​​in T5 and T3, respectively. Increasing the RO dose to 1 g/kg had a clear effect in improving the HSP70 levels ​​and achieving the highest values ​​in T4 and T5, respectively. The combined effect of RO at a low dose of 0.5 g/kg (T3) and ZF significantly increased HSP70 and HSP90 compared with the independent effect of RO (T2). The interactive impacts of adding RO at the high dose of g/kg (T5) and ZF remarkably increased HSP90 compared to all groups and HSP70 compared to T1, T2, and T3.

#### GH and IGF1

**Fig. 7:**
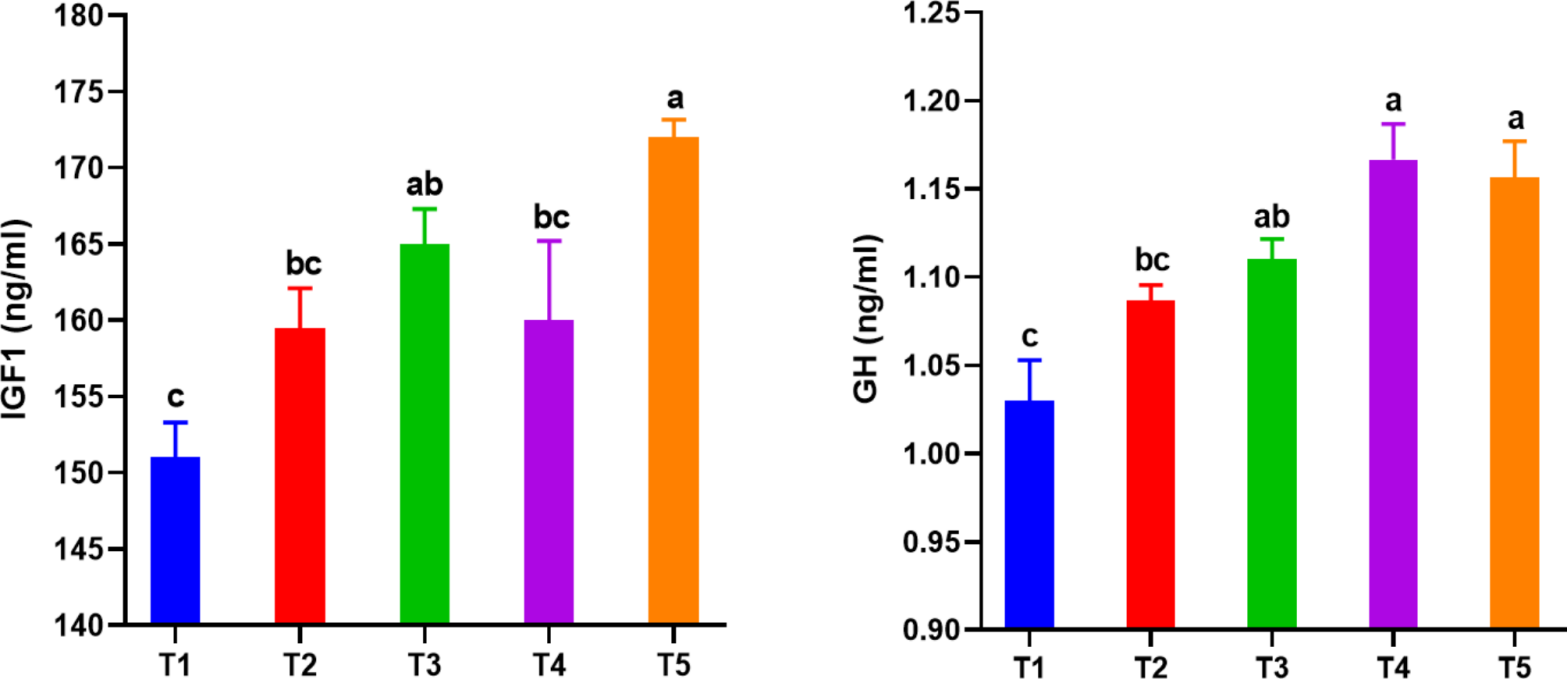
Growth hormone (GH) and insulin-like growth factor 1 (IGF1) in *Chelon ramada* fed a basal diet containing varying levels of rosemary oil and/or Zymogen Forte during a 75-day rearing trial using brackish groundwater. Data are presented as mean ± SEM of 3 replicates, where (T_1_) = fish fed BD without RO or ZF (control); (T_2_) = fish fed the BD containing 0.5 g of RO kg^− 1^; (T_3_) = fish fed BD containing 0.5 g of RO and 1 g of ZF kg^− 1^; (T_4_) = fish fed BD containing 1.0 g of RO kg^− 1^; and (T_5_) = fish fed BD containing 1 g of RO and 1 g of ZF kg^− 1^; BD = basal diet, RO = rosemary oil; ZF = Zymogen Forte

The independent and combined effects of dietary RO and ZF on plasma GH and IGF1 are shown in Fig. 7. The interaction effect of ZF and RO at figboth minimum and maximum doses led to an improvement in serum IGF1 compared to the control group, recording a significant (*P < 0.05*) value at T5 that did not differ significantly from T3. No major (*P > 0.05*) differences were recorded between T1, T2, and T4 concerning IGF1, despite the improvement in T2 and T4. The addition of RO at a high dose of 1 g/kg (T4 and T5) resulted in a significant improvement in plasma GH that was not significantly different from T3, which had the interaction of ZF and RO at a low dose of 0.5 g/kg diet. Adding RO at a low dose of 0.5 g/kg (T2) did not lead to significant (*P < 0.05*) enhancement in plasma GH compared to T1 (control group).

### Histopathological findings

**Fig. 8:**
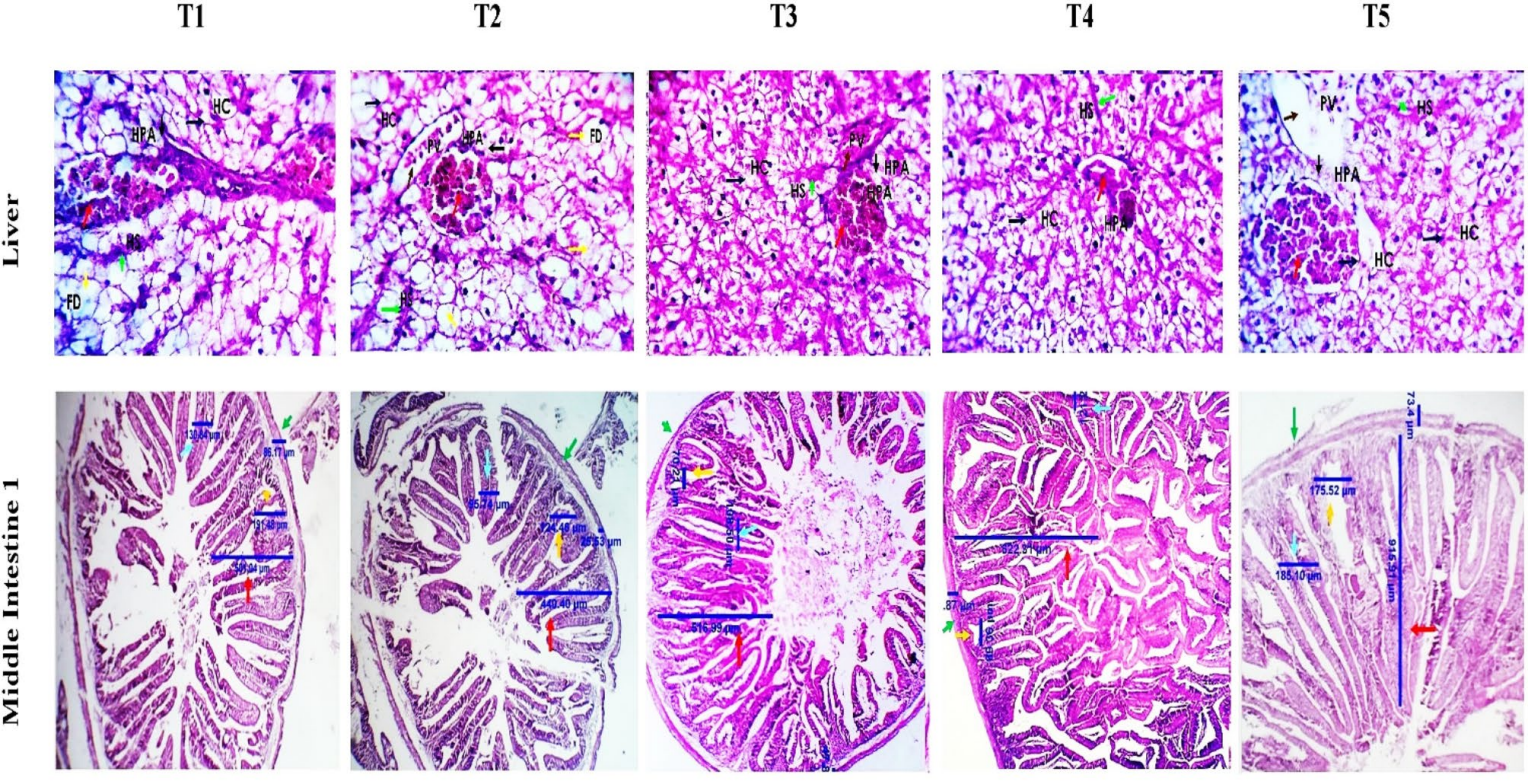
Histo-morphological changes in the liver, and middle intestine of *Chelon ramada* fed a basal diet containing varying levels of rosemary oil and/or Zymogen Forte for a 75-day rearing trial using groundwater. Photomicrographs showing fish liver groups of the present study (1–5) with apparently normal lobular arrangement, hepatocytes (HC, dark blue arrows) hepatic sinusoids (HS, green arrows) and portal area structures (PA, light blue arrows) enclosing a branch from the portal vein (PV, brown arrows). Fat droplets and globules (FD, yellow arrows) macro- and micro-steatosis, and portally aggregated melano-macrophages are outstanding in groups 1 and 2. Such changes appear minimal in groups 3, 4, and 5. Hepatocellular cytoplasmic basophilia (higher anabolic ribosomal contents) is seen in groups 3, 4, and 5. Photomicrographs of the middle intestine (1, 2, 3) demonstrating the histo-morphological changes in the middle intestinal segment of treatment groups (1–5), with a variable dimensional change regarding villous length (red arrows), villous width (blue arrows), crypt of Lieberkühn length (orange arrows), muscular coat thickness (green arrows) and goblet cell populations (yellow arrows). Liver and middle intestine stained with H&E X 100, 200, 400. X100, bar = 100 μm. Liver 1 and liver 2 with magnification 100 and 200X. Middle intestine 1, 2, 3 with magnification 100, 200, and 300X. Treatments: (T_1_) = fish fed BD without RO or ZF (control); (T_2_) = fish fed the BD containing 0.5 g of RO kg^− 1^; (T_3_) = fish fed BD containing 0.5 g of RO and 1 g of ZF kg^− 1^; (T_4_) = fish fed BD containing 1.0 g of RO kg^− 1^; and (T_5_) = fish fed BD containing 1 g of RO and 1 g of ZF kg^− 1^; BD = basal diet, RO = rosemary oil; ZF = Zymogen Forte

The histopathological view of *C. ramada* liver and mid-gut is displayed in Fig. 8. Photomicrographs of fish livers in the five groups showed a normal lobular arrangement, hepatocytes, hepatic sinusoids, and portal area structures containing a branch from the portal vein. Fat droplets and globules, macro- and micro-steatosis, and partially aggregated melano-macrophages are remarkable in groups 1 and 2, while such changes appear minimal in groups 3, 4, and 5. Hepatic cytoplasmic basophils (high structural ribosome contents) are observed in groups 3, 4, and 5.

Microscopic examination of the midgut in *C. ramada* showed that the histomorphological dimensions of the fish intestine are significantly affected by the addition of RO and ZF to the diet (Table 5; Fig. 8). The interaction effect between RO (1 g/kg) and ZF led to a significant (*P < 0.05*) increase in the villi length and width (T5). Furthermore, the interactive impacts of RO (0.5 g/kg) and ZF resulted in a considerable increase (*P < 0.05*) in the number of goblet cells (T3) compared to all groups except for group T2, which showed the same increase in goblet cell number. The higher dose of RO (1 g/kg) at T4 resulted in significant improvements in villus length and width compared to the lower dose (0.5 g/kg) at T2. On the other hand, the lower dose of RO at T2 significantly enhanced goblet cell numbers compared to the higher dose at T4.

**Table 5:** Histomorphometric changes (goblet cell count, villi length and width, crypts of lieberkühn length and muscle coat thickness) in the midgut of *Chelon ramada* fed a basal diet containing different levels of rosemary oil and/or Zymogen Forte for a 75-day rearing trial in brackish groundwater

Parameter*	Treatments					*P*-Value		
	T1	T2	T3	T4	T5	RO	ZF	RO*ZF
Villi length, µm	485.6 ± 8.37^**c**^	435.0 ± 3.61^**c**^	510.4 ± 4.79^**c**^	609.6 ± 10.70^**b**^	815.6 ± 57.03^**a**^	0.000	0.001	0.055
Villi width, µm	123.58 ± 2.86^**b**^	92.28 ± 2.12^**d**^	105.86 ± 1.42^**c**^	115.85 ± 3.08^**bc**^	176.11 ± 6.16^**a**^	0.000	0.000	0.000
C. length, µm	185.05 ± 3.68^**a**^	119.88 ± 2.5^**c**^	69.41 ± 1.50^**e**^	83.85 ± 3.13^**d**^	171.92 ± 2.35^**b**^	0.000	0.000	0.000
M.C. Thick, µm	81.78 ± 2.52^**a**^	24.01 ± 1.23^**d**^	45.35 ± 1.47^**c**^	47.60 ± 1.91^**c**^	70.75 ± 1.45^**b**^	0.000	0.000	0.571
GCC	22.67 ± 1.76^**b**^	26.0 ± 2.08^**ab**^	31.33 ± 2.60^**a**^	16.0 ± 1.15^**c**^	22.67 ± 0.88^**b**^	0.001	0.011	0.723

Data are presented as mean ± SEM of 3 replicates, with different letters in the same row presenting significant differences (*p* < 0.05). (T_1_) = fish fed BD without RO or ZF “control”; (T_2_) = fish fed the BD containing 0.5 g of RO kg^− 1^; (T_3_) = fish fed BD containing 0.5 g of RO and 1 g of ZF kg^− 1^; (T_4_) = fish fed BD containing 1.0 g of RO kg^− 1^; and (T_5_) = fish fed BD containing 1 g of RO and 1 g of ZF kg^− 1^; BD = basal diet, RO = rosemary oil; ZF = Zymogen Forte, RO*ZF = Interaction; C. length = crypts of Lieberkühn length; M.C. Thick = muscle coat thickness; GCC = Goblet cell count

## Discussion

Expanding desert mariculture using GW is a key solution to overcome acute freshwater shortage and sustain aquaculture development. Water source, and thus water parameters, have fundamental influences on all aspects of the performance of cultured aquatic species [8, 59, 60]. Therefore, introducing some nutritional or non-nutritional additives that promote diet quality may lead to improved culture water parameters, positively affecting the fish performance, welfare and health of the internal organs (such as the liver, which may accumulate toxic elements hundreds of times higher than what can be evaluated in the surrounding environment) [14].

In our study, there was an overall improvement in water quality parameters (NH_4_, NH_3_ and NO_2_) in all tested groups compared to the control group as a result of the inclusion of RO and RO + ZF in fish diets, with the interaction between ZF and RO at T3 and T5 showing the most desirable declines. These decreases can be linked to improved protein metabolism and therefore metabolism in the fish body, leading to lessened NH_3_ secretion and lowered nitrogen by-product accumulation in culture tanks [1, 61]. In addition, the stimulatory effect of RO and RO + ZF may promote the utilization of dietary protein in fish growth rather than in another metabolic process, resulting in the outcomes of decreased NH_3_ and NO_2_. Moreover, the antimicrobial effects of RO against pathogenic bacteria [29] combined with the active ingredients in ZF such as digestive enzymes, anti-flatulence agent and others promoted gut health and digestive processes leading to reduced NH_3_ release [62]. In line with these findings, declines in NH_3_ levels were documented with the Nile tilapia fed 2% thyme essential oil [63], and *C. ramada* fed RO and RO + amylase and lipase enriched diets [1]. Similarly, there was a minor decrease in NH_4_ levels observed when seabass were given a meal containing 1% thyme, rosemary, and fenugreek extract [61]. However, there was no notable decrease in NH_3_ levels detected in the nursery water of great sturgeon (*Huso huso*) that were fed diets enriched with RO [64]. This discrepancy may be due to differences in fish species, fish age, water standards, culture systems employed, and techniques. The lower number of pathogenic bacteria identified in the culture water in the tested groups compared to the control group is due to the antimicrobial action of RO in inhibiting the growth of pathogenic microbes [29, 65, 66]. In addition, RO has been confirmed to have anti-mycotoxin influences [29] and antifungal activity due to its phenolic compounds [67] and antibacterial efficacy against both Gram-positive and Gram-negative bacteria [68]. Interestingly, the complementary impact of ZF with RO boosted this property, resulting in a greater lowering of pathogenic bacteria counted at T5 and T3 compared to all groups. Compounds in RO that deliver antimicrobial impacts have been specified as α-pinene, camphor, verbenone, 1,8- cineole, α-terpineol, phenolic diterpenoid, and carnosic acid [68]. In line with our findings, RO exhibited marked antimicrobial activity against *Staphylococcus aureus*, *Escherichia coli*, *Klebsiella pneumoniae* and *Bacillus subtilis*, and the most susceptible strain was *S. aureus* [65]. Moreover, tilapia fed with *Rosmarinus officinalis* -supplemented diets and injected with a highly contagious pathogen (*Streptococcus iniae*) showed significantly higher resistance and lower mortality rates than those fed control diets [69]. Furthermore, RO exhibited inhibitory characteristics against the growth of *Flavobacterium psychrophilum*, the pathogenic microorganism responsible for bacterial cold-water illness in salmonid aquaculture facilities [70]. In addition, the inclusion of 0.5% RE in the diet improved tilapia resistance to diets contaminated with aflatoxin B1 [29]. In the same context, carnosic acid (which accounts for 40% of RE) modified the gut microbiota, promoted probiotics and functional bacteria in high-fat diet-induced obese mice, as well as inhibited bile acid-metabolizing bacteria [66]. The differential impact of RO and RO + ZF on the computed bacterial quantities in *C. ramada* water can be ascribed to the resistance exhibited by each species and the robustness of the outer cell envelope, which serves as a shield against all external influences [65].

Therapeutic plants and their derivatives are utilized in aquaculture because they have a beneficial impact on growth by enhancing protein synthesis, boosting immunity, improving intestinal secretions by directly killing harmful bacteria in the gut, and increasing the production of digestive enzymes [51, 64, 71]. Herein, the addition of RO (low and high dose) or RO + ZF, significantly improved GH and IGF1 activity in *C. ramada* grown with GW, resulting in improved growth parameters and survival rate. The mechanism by which RO or RO + ZF stimulates GH and IGF1 is unclear and has not been discussed previously nor in this study. However, this could be attributed to the enhanced well-being of the fish liver, which is the primary source of IGF1 in vertebrate animals [72]. GH is a 22-kDa protein of the pituitary root that has maintained a pleiotropic impact throughout vertebrate evolution [73]. IGF1 is a 7-kDa mitogenic polypeptide of 70 amino acids closely linked to structural proinsulin that plays a critical role in regulating development and growth by mediating the action of GH [72, 74]. GH levels in fish plasma alter seasonally in reply to environmental conditions like photoperiod and temperature [75], while IGFI levels are regulated by GH and directly related to nutritional factors [76]. Hence, the GH–IGFI axis may deliver an integrated sign concerning season, temperature, and food supply [74, 76]. The role of GH in fish growth, water adaption, reproduction, and immunological function has now been confirmed, with the liver being the primary tissue where GH directly acts [72, 73]. Moreover, stimulation of GH by an exogenous factor can promote further growth under many conditions in healthy animals, as GH is one of the most critical factors controlling body growth [74, 77] and this most likely occurred because of the addition of RO and RO + ZF. Here, variation in plasma GH and IGF1 between groups is positively associated with variation in growth performance, reinforcing the earlier idea suggested that IGF1 prevalence may serve as a physiological marker of growth rate in fish [74, 78]. In line with the current findings, Juvenile olive rockfish showed faster growth under a 4% daily feed ration associated with higher plasma IGF1 levels than fish under a 1% daily ration (starving) [74]. Furthermore, there was a correlation between higher levels of GH in the blood and enhanced growth. Additionally, increased levels of immunoreactive IGFI, likely produced by the liver in response to GH activity, were also positively associated with growth in gilthead sea bream (*Sparus aurata*) [79]. The improved growth indices for fish-fed diets treated with RO and RO + ZF are in accord with those reported for the Nile tilapia [27, 29], common carp [28] rainbow trout (*Oncorhynchus mykiss*) [71], and *L. ramada* [1] when fed diets supplemented with varying proportions of rosemary extract. Moreover, the administration of exogenous zymogen at 2 and 4% improved the survival and growth rate of grey mullet *Liza ramada* larvae [30], and it was also introduced as an anti-flatulent in the diet of *Dicentrarchus labrax* larvae [80]. The previous authors ascribed these enhancements in fish growth and feed efficiency to the active substances (carnosol and rosmanol) discovered in this plant, which have been acknowledged as digestive stimulants, immunostimulants, potent antioxidants, and antimicrobial agents against pathogenic bacteria in the intestine. On the other hand, there was no observed enhancement in the growth of fish and the efficiency of feed utilization while using *S. aurata* [81], seabass [61] and *H. huso* [64] due to the inclusion of rosemary in the diet. This discrepancy may be because of various fish species, feeding periods, rosemary origins, part of the plant used, and rearing techniques applied. The results obtained for GH, IGF1, and growth parameters show that ZF addition enhanced the action of RO, resulting in higher values ​​of these parameters in T3 and T5.

As promising nutritional supplements in aquaculture, the use of herbal derivatives aims to promote fish immunity and boost their performance when reared in such desert groundwater. Immune responses in fish are mediated by a variety of cells and secreted soluble mediators which work in synergistic form for full defense [64, 82]. WBC (leucocytes) including lymphocytes (T cells, B cells), and phagocytes (monocytes and neutrophils) serve as the backbone of all immune responses [28]. Lymphocytes not only produce antibodies via distinct protective mechanisms but also exhibit macrophage activity which means the fish’s immune defense system is improved [64]. In the current study, the interactive effect of ZF and RO (high dose) at T5 showed the highest increase in leukocyte count while the highest number of lymphocytes was reported at T3 (ZF + low dose of RO) and T4 (high dose of RO). In agreement with these findings, earlier investigations demonstrated increased WBC counts in fish-fed rosemary leaf powder [28], rosemary essential oil [64] therapeutic herbs and their active compounds [17, 29, 83]. Besides leucocytes and lymphocytes, the complement system (CS) plays a crucial role in alerting the host of the existence of potential pathogens, as well as in their resistance [29]. CS activation contributes greatly to the orchestration and evolution of an acquired immune response as it is one of the humoral parts of the innate immune defense that optimizes phagocytosis, and cytolysis, and regulates inflammation [29, 84]. C3 and C4 are crucial for the activation of the complement system. They serve as essential components of the C3 convertases in the alternative and classical pathways, respectively, while not having catalytic activity [85]. Herein, the high values of C_3_ and C_4_ recorded with the fish-fed RO and RO + ZF-supplemented groups, particularly at T3 and T5, indicate that the inclusion of RO and ZF in *C. ramada* diet enhanced their innate immunity. These findings are consistent with those reported for Mozambique tilapia [86] and the Nile tilapia [29], where oral administration of rosemary improved disease resistance, CS activity, and general immunity.

In addition, innate immune markers such as TP, ALB, GLO, and IgM are key players in the genesis of the immune response [28, 87]. TP is a crucial clinical measure of nutritional status, stress levels, the effectiveness of the immune system, the well-being of marine species, and the ability to maintain proper blood pH and osmotic pressure in the face of environmental challenges [18, 88]. ALB and GLO are two essential parts of TP and the main source of immunoglobulin production [64, 89]. ALB handles lipids transport and general metabolism [90, 91]. The IgM superfamily consists of immunoglobulins, sometimes known as antibodies, which play a crucial role in adaptive immune responses. IgM is the predominant immunoglobulin in fish serum and has traditionally been considered the earliest class of antibodies in response to viruses and pathogens. In this humoral defensive mechanism mediated by Ig (immunoglobulin), the complement system is activated, viruses and toxins are neutralized, and phagocytes are encouraged to eliminate infections [18, 91]. The elevated concentrations of plasma TP, ALB, and IgM in fish from the supplemented groups are believed to be linked to a more robust innate immune response, suggesting enhanced performance of protein-producing organs such as the liver [18, 64]. In agreement with these results, higher levels of TP, ALB, and GLO were observed in common carp-fed diets supplemented with rosemary extract and in *Huso huso*-fed diets supplemented with RO [64], and higher levels of TP and GLO were observed in the Nile tilapia fed diet contaminated with aflatoxin B1 and supplemented with 0.5% rosemary [29]. Likewise, oral administration of eucalyptol to common carp significantly attenuated copper-induced decreases in lysozyme, ACH50 and total Ig [92], while lavender oil fed to seabass showed increased levels of TP, ALB, GLO, and IgM [17]. The mechanism by which RO or RO + ZF promotes the immune system of fish is not quite clear. However, this may be due to enhancing liver health and function, as this organ is the site of many proteins synthesis [52]. In addition, rosemary extract or its active components have been found to have anti-inflammatory, hepatoprotective, antidiabetic, antinociceptive, anticancer, and antioxidant activity in experimental animals and humans [1, 28, 29, 64]. Moreover, ZF has many active ingredients (digestive enzymes, antiflatulence agent, ox-bile extract, hemicellulase, pepsin, dimethylpolysiloxane and vitamin B1) that may stimulate these immune markers in fish.

The antioxidant system, mostly composed of SOD, CAT, and GPx, plays a crucial role in preserving fish health and safeguarding live cells against free radicals, excessive inflammation, and apoptosis [17, 93, 94]. When exposed to stressors, the antioxidant system may malfunction, resulting in reduced activity of SOD and CAT. This leads to the oxidation of the organism’s biological substances by reactive species and free radicals, resulting in the formation of numerous end products [28, 29]. Multiple stressors, including pollution, ambient temperature, stocking density, handling, water supply, water physicochemical characteristics, and sorting, constantly impact fish in an aquaculture system [21, 51]. Increases in cortisol (COR), glucose (GLU), and creatinine (CRE) levels are recognized as primary and secondary reactions to stress [28, 95, 96]. The rise in these indicators is a sign of the existence of adaptive strategies to provide energy demands to cope with stress [89, 95]. Our results here indicate that dietary intake of RO and RO + ZF improved antioxidant activity and suppressed COR, CRE and GLU elevations in *C. ramada* grown in GW. The improved antioxidants (TAC, CAT, SOD and GPx) and suppressed COR, CRE and GLU levels could be attributed to the anti-inflammatory and hepatoprotective impacts of RO along with the anti-flatulence effect of ZF. Also, improved water quality parameters as evidenced by lower NH_3_ and No_2_ levels and pathogenic bacterial load, resulted in lower stress in the culture water of treated groups compared to the control group, leading to lower COR, CRE and GLU levels and increased antioxidant activity. In line with these outcomes, plasma GLU levels in *H. huso* were notably declined by using RO and oxytetracycline compared to the reference group [64]. The reduction in plasma glucose (GLU) levels in fish treated with therapeutic herbs was attributed to the enhancement of the antioxidant defense system, resulting in improved insulin synthesis in the pancreas [64, 97]. In the same vein, dietary rosemary extract administration reduced COR and GLU levels and mitigated the adverse effects of crowding stress on common carp [28]. Likewise, dietary cineole attenuated the increase in serum COR in rainbow trout exposed to crowding stress for 14 days [98], and oral management of eucalyptol markedly lowered plasma COR and GLU levels in common carp, after copper intoxication [92].

Heat shock proteins (HSPs) are a group of stress proteins that are produced in reaction to both living and non-living stress factors [99]. HSPs are highly maintained, both in structure and amino acid sequence, and depending on protein molecular size they are typically classified into families such as HSP100, HSP90, HSP70, HSP60, and the small HSPs [100, 101]. Heat shock proteins (HSPs) are produced in normal physiological conditions and in reaction to stress. They play a role in the folding and assembly of protein chains, repairing damaged proteins, immunological responses, cell death, and other physiological processes [100, 102]. HSPs have been observed to increase the ability to withstand stress and have a wider impact on several parts of the immune system and the inflammatory response in numerous aquatic organisms [100]. In fish, as in mammals, HSP90 and HSP70 are associated with cytoprotection, cell survival and immune rejoinders that exert a protective role [103]. In addition, HSP90 is an inducible chaperone that is associated with stress-induced cytoprotection [103, 104]. HSP70 is crucial in assisting unstressed cells, and its inducible form rapidly increases during cellular stress. It can be located in both the cytoplasm and extracellular compartments, where it contributes to innate immunity [104, 105]. Increased levels of HSP70 and HSP90 (involved in rebuilding damaged proteins in the body’s cells) in fish fed with RO and RO + ZF compared to the control group could be an indicator of increased stress tolerance of fish, as fish grow in GW that is quite different from fresh and marine water. This hypothesis is supported by the findings of Cara et al. [102] that enhanced HSP70 and HSP90 levels were associated with clear stress tolerance in food-deprived *Sparus aurata* and *Oncorhynchus mykiss* larvae. Additionally, it was revealed that the presence of HSP70 and HSP90 during the initial stages of fish development might serve as an indicator of nutritional stress. Furthermore, the augmentation of HSPs levels can be utilized as a method to enhance the ability to withstand stress during the rearing of larvae [102]. No aforementioned studies have investigated the effect of rosemary plant, its derivatives, or any other essential oil on HSP_S_ in fish. However, the higher levels of HSP70 and HSP90 in the RO and RO + ZF groups may be attributed to the presence of bioactive substances in RO and ZF that enhanced their activity to rebuild damaged proteins in fish cells. This hypothesis is supported by previous studies revealing that glutamine increased HSP70, HSP25, and HSP90 in rats [106, 107], L-arginine increased HSP70 and HSP90 in rats [108], whey protein hydrolysate increased HSP70, and HSP90 in rats [109], chia oil with high-fat-high-fructose increased HSP70 and HSP25 in rats [110, 111], and curcumin increased HSP30, HSP70 in *Xenopus laevis* [111, 112]. Likewise, increasing salinity above 25 mg/l increased the expression of HSP70 in the gills and kidneys of *Srikandi tilapia* [55]. Also, the value of HSP70 in both members of silver rasbora (*Rasbora argyrotaenia*) increased with increasing exposure to sublethal concentrations of organophosphate pesticides [113]. Our results here support the idea that the dietary addition of RO and ZF led to improved HSP70 and 90 activity of *C. ramad* grown in saline GW, thereby boosting their performance and overall health. However, further research is needed on the limitations (overdoses used, economic feasibility, quality of treated fish, etc.) and future research directions (role in rebuilding damaged proteins, upgrading the innate immune system and stress resistance) of using RO and RO + ZF on aquatic animals.

The liver is a vital organ that plays a role in energy metabolism and storage, food digestion (by synthesizing bile), and protein synthesis such as coagulation factors, vitellogenin, and hormones including IGF1 and thrombopoietin [114]. However, in fish, the liver is also one of the most typical targets for both cytotoxicity and tumorigenicity, and pollutants such as HM can be condensed in the fish liver, even at very low concentrations [14, 114]. Therefore, histopathological assessment of the livers and intestines of *C. ramada* grown in GW may demonstrate the potential ameliorating effects and safe use of RO or RO + ZF in aquaculture. Microscopic assessment indicated no pathological changes in the liver tissue of *C. ramada* in all groups tested. However, the fish livers in groups T3, T4, and T5 were healthier with hepatocellular cytoplasmic basophilia compared with those in groups T1 and T2, which showed fat droplets and globules, macro- and micro-steatosis, and portal aggregated melanomacrophages (Fig. 8). The hepatoprotective effect of RO at a dose of 1 g/kg (T4) and RO + ZF (T3 and T5) may be due to the antioxidant properties of RO combined with the anti-flatulent and digestive-stimulating effects of ZF, which led to the prevention of lipid peroxidation in the liver cell wall [28] and thus reduced inflammatory properties [17, 26]. Consistent with this finding, histopathological markers of the Nile tilapia livers exposed to aflatoxin B1 improved significantly up to normal levels when fish were fed with diets supplemented with 0.25–0.5% rosemary [29]. The improved intestinal health (villus length and villus width) and increased goblet cell numbers in fish-fed RO + ZF-supplemented diets may be due to the stimulated production of endogenous enzymes that promote cholecystokinin secretion and pancreatic exocrine secretion, thus modifying and stimulating gastrointestinal physiology and facilitating digestion and absorption of feed and nutritious accompaniments. Furthermore, rosemary has therapeutic properties that include anti-inflammatory, antibacterial, anticoagulant, antinociceptive and antioxidant activities [25] while ZF contains active compounds including digestive enzymes and an anti-flatulence agent. Therefore, the interaction between these active components in RO and ZF may serve to keep the intestines healthy and free of pathogens, hence improving these morphological measurements. These outcomes concur with those reported for *L. ramada* fed RO and RO + amylase-lipase supplemented diets [1], seabass-fed dietary lavender oil [17] and common carp-fed dietary oregano essential oil [115].

## Conclusion

Derivatives and extracts of medicinal plants are utilized in aquaculture for multiple objectives, such as promoting development, enhancing immunity, and providing functional additives like resistance to environmental stress. The experiment revealed that the addition of 1 g RO or 0.5–1 g /kg RO + ZF to a *C. ramada* diet has several advantages, such as decreased NH_3_ secretion, NO_2_ accumulation, and pathogenic bacterial load in GW utilized in culture tanks. Moreover, notable enhancements were identified in the growth rates and related hormones, feed efficiency, immunological function, and overall health of the fish. These gains were demonstrated by advancements in biochemical blood markers and the condition of internal organs (intestine and liver). Considering the improved immune function and greater performance in T5 and T3 groups, it is recommended to include RO at a level of 0.5–1 g/kg with ZF in the diet of *C. ramada* when cultivated in GW. Diet augmentation with RO and RO + ZF exhibited improvement in HSP90 and HSP70 which are responsible for the repair of damaged proteins, cell survival, immune rejoinders, and stress-induced cellular protection, opening up potential applications for the aquaculture industry under challenging farming conditions. Additional research is necessary to establish a comprehensive understanding of the impact of supplements produced from medicinal plants in enhancing the immune response and stress resilience in fish raised under tough culture conditions and their suitability for different marine species in the context of using GW for desert mariculture development.

## Data Availability

Availability of data and materials: the datasets developed during and/or examined during the present investigation are available from the corresponding author upon request.

## Declarations

### Guidelines

All methodologies were performed following relevant guidelines and regulations.

### Arrive guidelines

The authors affirm that the study was conducted in adherence to the ARRIVE guidelines.

## Abbreviations

GW: Groundwater
RO: Rosemary oil
ZF: Zymogen forte™
BD: Basal diet
HSP70: Heat shock proteins 70
HSP90: Heat shock proteins 90
NH4: Ammonium
NH3: Ammonia
NO2: Nitrite
HM: Heavy metals
TT: Thousand tons
RE: Rosemary extract
MRSAR: El-Max Research Station for Applied Research
TP: Total protein
ALB: Albumin
GLO: Globulin
IgM: Immunoglobulin
CRE: Creatinine
COR: Cortisol
GLU: Glucose
C3: Complement 3
C4: Complement 4
CAT: Catalase
SOD: Superoxide dismutase
MDA: Malondialdehyde
TAC: Total antioxidative capacity
GH: Growth hormone
IGF1: Insulin-like growth factor 1
ELISA: Enzyme-linked Immunosorbent Assay
